# Hybrid Improved Bird Swarm Algorithm with Extreme Learning Machine for Short-Term Power Prediction in Photovoltaic Power Generation System

**DOI:** 10.1155/2021/6638436

**Published:** 2021-08-26

**Authors:** Dongchun Wu, Jiarong Kan, Hsiung-Cheng Lin, Shaoyong Li

**Affiliations:** ^1^College of Electrical Engineering, Yancheng Institute of Technology, Yancheng 224051, China; ^2^Department of Electronic Engineering, National Chin-Yi University of Technology, Taichung 41170, Taiwan; ^3^School of Civil Engineering, Lanzhou University of Technology, Lanzhou 730050, China

## Abstract

When a photovoltaic (PV) system is connected to the electric power grid, the power system reliability may be exposed to a threat due to its inherent randomness and volatility. Consequently, predicting PV power generation becomes necessary for reasonable power distribution scheduling. A hybrid model based on an improved bird swarm algorithm (IBSA) with extreme learning machine (ELM) algorithm, i.e., IBSAELM, was developed in this study for better prediction of the short-term PV output power. The IBSA model was initially used to optimize the hidden layer threshold and input weight of the ELM model. Further, the obtained optimal parameters were input into the ELM model for predicting short-term PV power. The results revealed that the IBSAELM model is superior in terms of the prediction accuracy compared to existing methods, such as support vector machine (SVM), back propagation neural network (BP), Gaussian process regression (GPR), and bird swarm algorithm with extreme learning machine (BSAELM) models. Accordingly, it achieved great benefits in terms of the utilization efficiency of whole power generation. Furthermore, the stability of the power grid was well maintained, resulting in balanced power generation, transmission, and electricity consumption.

## 1. Introduction

The ecological environment on earth is significantly threatened due to the large consumption of fossil energy [1]. Therefore, renewable energy resources have been exploited and widely applied to reduce nonrenewable energy consumption globally [2–4]. Among the various sources of renewable energy, solar energy has attracted a lot more attention in recent years [5, 6]. However, Shafi et al. [7] indicated that photovoltaic (PV) power generation might suffer some drawbacks, such as randomness and unpredictability. The imbalance between supply and demand is bound to lead to a long-term inefficiency in the power distribution. Essentially, weather conditions are found to be the key factor in intermittent and unstable PV power generation. Consequently, their impact on the PV power generation forecast must be considered [8]. For the power forecast study, the PV system can be classified into ultra-short, short, medium, and long periods [9]. The ultra-short power forecast generally takes 0–4 hours, while the short power forecast takes 1–3 days. The medium- and long-term power predictions usually take one week and one month, respectively. Among the four time periods, the short power forecast plays a crucial role in power dispatching [10–12].

At present, the primary method employed for ultra-short-period power forecast in the PV output power is the satellite nephogram used to predict the light intensity for the subsequent few hours [13, 14]. Short-term power prediction collects the meteorological data from the weather forecast for the upcoming 1–3 days [15]. However, medium- and long-term power predictions employ statistical methods based on historical data [16]. It is necessary to establish a PV power prediction model that can fit varying weather conditions for advanced technology development [17]. PV power prediction methods can be classified into indirect or direct prediction [18]. Indirect prediction approaches predict the solar radiation intensity from meteorological data and satellite images to obtain output power [19, 20]. Alternatively, the direct prediction method directly predicts the PV generation power based on the past power generation data, weather data, and future weather forecasts [21, 22]. In this study, a hybrid improved bird swarm algorithm (IBSA) with an extreme learning machine (ELM) model has been established to forecast PV power, based on the direct prediction principle. The main contributions of this study are as follows:

1. The optimization ability of traditional BSA was enhanced using improved BSA (IBSA).
2. IBSA was used to optimize the ELM model. As a result, the PV power generation forecast based on ELM model was able to achieve better performance.
3. The power generation scheduling could be arranged more reasonably and the safety in the electrified wire netting was therefore ensured.

## 2. Literature Review

Currently, various algorithms that use direct prediction methods have been reported to predict PV power generation [23, 24], which are mainly classified into linear forecast methods, nonlinear forecast methods, and mixed forecast methods [18]. The time series prediction method and time trend extrapolation method belong to linear forecast methods [25, 26]. However, linear forecast methods may produce a large deviation when there are great changes in the external environment such as weather or temperature [27, 28]. Nonlinear forecast methods are more suitable for PV power output prediction, which generally employ artificial neural network (ANN), Gaussian process regression (GPR), extreme learning machine (ELM), support vector machine (SVM), and more [29, 30].

ANN is a mathematical model that imitates the biological neural network. Nespoli et al. [31] used two ANNs to predict the output power of the PV system. On sunny days, the results exhibit a good and stable prediction performance. However, on cloudy days, the forecasting results became evidently worse. Rocha et al. [32] adopted ANN to predict the PV power using temperature and radiation data collected from different locations, revealing that the experimental results were close to the actual values. However, the prediction accuracy may be sensitive to weather conditions. Khan et al. [33] proposed a hybrid power forecasting model for PV plants, using back propagation neural network (BP) with an air quality index (AQI). Although the prediction error was small in the haze weather, it requires tremendous data and computational time. On the other hand, GPR is an extension model of a multivariate Gaussian distribution in machine learning, signal processing, and more. For instance, Sheng et al. [34] applied the GPR method to predict PV power. Cheng et al. [9] proposed an improved sparse GPR model with an improved least square support vector machine (SVM) model for PV output power prediction.

SVM is known for its superior capability of structural risk minimization and operation speed [35, 36]. Wang et al. [37] combined SVM with the K-nearest neighbors (KNN) algorithm for PV power generation prediction, demonstrating that the accuracy rate of SVM was higher using fewer samples; however, KNN was found to be more accurate than SVM when the sample number was sufficient. Aprillia et al. [38] proposed a PV generation prediction strategy which combined a convolution neural network (CNN) with a salp swarm algorithm (SSA), i.e., CNN-SSA. When compared to long short-term memory (LSTM) method combined with SSA (LSTM-SSA), and the SVM method combined with SSA (SVM-SSA), CNN-SSA exhibited superior performance regarding PV power generation prediction. Yang et al. [39] used the grey relevance theory to find the major elements that may affect the consumption of clean energy. Moreover, the least squares support vector machine (LSSVM) was optimized by evolutionary game theory (EGT) and the adaptive differential evolution (ADE) algorithm. It was confirmed to work well in the generalization ability and prediction effect. VanDeventer et al. [22] developed a short-term forecast model using SVM with genetic algorithm (SVMGA). The prediction error received was found to be smaller than that in traditional SVM. However, it is sensitive to the parameter values of the model and is unsuitable for training large-scale samples.

Fast learning speed and good generalization are the two main advantages of ELM [40, 41]. Liu et al. [42] used ELM to predict the short-period PV power generation. The weights of the ELM algorithm were updated via particle swarm optimization (PSO) algorithms. Although it has a desirable forecast effect, the computational complexity was increased. Zhou et al. [41] proposed a prediction model using similar day analysis and genetic algorithm to optimize ELM (SDA-GA-ELM) and enhance the forecast precision. The results of its application on PV output power prediction showed that the SDA-GA-ELM model improves the prediction accuracy of ELM. Luo et al. [43] combined deep learning with a stacked ELM model. The generalized entropy criterion was then used to improve the prediction efficiency. The results revealed its prediction effect to be better than ANN and ELM models.

Intelligent algorithms have been widely applied to improve the performance of prediction models. For instance, the PSO algorithm is a random search algorithm based on group cooperation. Behera et al. [42] proposed PSO to optimize the prediction performance of the ELM model. The chicken swarm optimizer (CSO) algorithm can simulate the hierarchical system and activity behavior of a swarm. Liu et al. [44] used the CSO to optimize the key parameters of the ELM model, thus improving the prediction of the output power from the PV power generation. The ant colony optimization (ACO) algorithm is a highly innovative metaheuristic algorithm inspired by ant foraging behavior. Li et al. [45] applied ACO algorithms to optimize the BP neural network for the prediction of the number of people participating in the make-up exam. The results revealed that the optimized BP neural network has a desirable prediction performance. The bird swarm algorithm (BSA) proposed by Meng et al. [46] is a global optimization algorithm used to simulate bird swarm behavior. Unfortunately, it is easy to fall into a local optimum at the high-dimensional multiextremum problem. In an advanced application, Alhassan and Wan Zainon [47] combined the Taylor series with BSA to optimize a classification model for the diagnosis of heart diseases. Besides, Huang and Sheng [48] proposed a hybrid bird swarm optimization algorithm to optimize the identification model in a thermal power plant. However, its operation time may be too long to be applied in a real circumstance.

## 3. The Proposed Prediction Model

### 3.1. Bird Swarm Algorithm and Improved Bird Swarm Algorithm

The bird swarm algorithm (BSA) was originally inspired from the flight and foraging behavior of birds in nature. Its processing rules are shown as follows [46, 49]:

1. Foraging behavior or vigilance behavior can be chosen by each bird.
2. The best information within the group and the individual can be recorded by each bird and shared within the group.
3. When the bird chooses the vigilance behavior, it will move to the center of the group, and there is competition during the process of movement.
4. The birds periodically migrate to the next location. Then, the producer can be selected using the best fitness. In the meanwhile, the scrounger can be selected using the worst fitness, and other producers or scroungers are randomly selected from the remaining birds.
5. Scroungers will follow producers to find food.

Let there be *M* birds. The time step is denoted as *t*, and the location of the *i*_th_ bird in the *j*_th_ dimension is *d*_*i*,*j*_^*t*^.

The rule (2) can be expressed as

(1) $$\begin{array}{c} d_{i,j}^{t+1}=d_{i,j}^{t}+R\left(b_{i,j}-d_{i,j}^{t}\right)\text{rand}\left(0,1\right)+D\left(z_{j}-d_{i,j}^{t}\right)\text{rand}\left(0,1\right), \end{array}$$

where rand(0,1) is an independent uniformly distributed random variable on the interval [0, 1]; *R* is the cognitive coefficient; *D* is the accelerated coefficient; *d*_*i*,*j*_^*t*^ represents the position at *t*; *b*_*i*,*j*_ is the best location of the *i*_th_ bird; and *z*_*j*_ is the best place.

When birds keep vigilance, their location is updated as follows:

(2) $$\begin{array}{c} d_{i,j}^{t+1}=d_{i,j}^{t}+E_{1}\left(d_{\text{mean},j}-d_{i,j}^{t}\right)\text{rand}\left(0,1\right)+E_{2}\left(b_{m,j}-d_{i,j}^{t}\right)\text{rand}\left(-1,1\right), \end{array}$$

(3) $$\begin{array}{c} E_{1}=e_{1}\exp\left(-\frac{f_{i}M}{\text{sum}f+\varepsilon }\right), \end{array}$$

(4) $$\begin{array}{c} E_{2}=e_{2}\exp\left(\left(\frac{f_{i}-f_{m}}{\left|f_{i}-f_{m}\right|+\varepsilon }\right)\frac{f_{m}M}{\text{sum}f+\varepsilon }\right), \end{array}$$

where *d*_mean,*j*_ represents the average position of the whole swarm in *j*_th_ dimension; rand(−1,1) is an independent uniformly distributed random variable on the interval [−1, 1]; *m* is a randomly selected positive integer (*m* ≠ *i*) where *m* ∈ [1, *M*] ≠ *i*; *b*_*m*,*j*_ represents the best previous position of the *m*_th_ bird and *f*_*m*_ is the optimal fitness value of the *m*_th_ bird; *e*_1_ and *e*_2_ are the constants between [0,2]; *f*_*i*_ represents the optimal fitness value of the *i*_th_ bird and sum*f* denotes the sum of the optimal fitness values in a bird swarm; and *ε* is an infinitesimal quantity.

The location update equation for producers is as follows:

(5) $$\begin{array}{c} d_{i,j}^{t+1}=d_{i,j}^{t}+d_{i,j}^{t}\text{rand}\left(0,1\right). \end{array}$$

The location update equation for scroungers is as follows:

(6) $$\begin{array}{c} d_{i,j}^{t+1}=d_{i,j}^{t}+\text{FL}\left(d_{m,j}^{t}-d_{i,j}^{t}\right)\text{rand}\left(0,1\right). \end{array}$$

In equation (6), FL represents the following coefficient, where FL ∈ [0,2]. *d*_*m*,*j*_^*t*^ denotes the position of the *m*_th_ bird.

The producer location is updated as follows:

(7) $$\begin{array}{c} d_{i,j}^{t+1}=a\left(t\right)d_{i,j}^{t}+d_{i,j}^{t}\text{rand}\left(0,1\right),\\ a\left(t\right)=\left(a_{\max}-a_{\min}\right)\cos\left(\frac{t}{t_{\max}}\pi \right)+a_{\min}, \end{array}$$

where *t* denotes the current iteration number and *t*_max_ is the maximum iteration number; *a*(*t*) represents the adaptive learning coefficient of the producer, which is the function of a cosine; and *a*_max_ and *a*_min_ are the maximum and minimum learning coefficients, respectively. In this study, *a*_max_ is set as 0.9, and *a*_min_ is set as = 0.4.

The relative change rate of the scrounger's fitness value *k* is defined as

(8) $$\begin{array}{c} k=\frac{\left|f_{i}-f_{b}\right|}{f_{b}}. \end{array}$$

In equation (8), the best fitness value of the *i*_th_ scrounger is *f*_*i*_ and the best fitness value of the bird swarm is *f*_*b*_.

Adaptive learning factor *Y*_*i*_^*t*^ in the *i*_th_ scrounger at *t* is used to update the scrounger location, and it is defined as

(9) $$\begin{array}{c} Y_{i}^{t}=\frac{1}{1+e^{-k}}. \end{array}$$

The location of the scrounger is updated as follows:

(10) $$\begin{array}{c} d_{i,j}^{t+1}=Y_{i}^{t}d_{i,j}^{t}+\text{FL}\left(d_{m,j}^{t}-d_{i,j}^{t}\right)\text{rand}\left(0,1\right). \end{array}$$

In BSA, it may have a disadvantage of slow convergence speed and low accuracy. Accordingly, the proposed IBSA model is developed to resolve this drawback. First, the adaptation values of the bird swarm are sorted when the groups fly to their end-point. Second, the first five ranking individuals are set as producers, and the last four individuals are selected as scroungers. The rest of the birds are randomly selected as producers or scroungers. The producer location is updated as follows:

(11) $$\begin{array}{c} d_{r,j}^{t+1}=\alpha \left(t\right)d_{r,j}^{t}+d_{r,j}^{t}\text{rand}\left(0,1\right),\;\left(1\leq r\leq 5\right). \end{array}$$

The location updated for scrounger is shown as follows:

(12) $$\begin{array}{c} d_{r,j}^{t+1}=Y_{r}^{t}d_{r,j}^{t}+\text{FL}\left(d_{m,j}^{t}-d_{r,j}^{t}\right)\text{rand}\left(0,1\right),\;\left(M-4<r\leq M\right), \end{array}$$

where *d*_*r*,*j*_^*t*^(*r* ∈ [1, *M*]) is the individual position of the swarm after sorting and *Y*_*r*_^*t*^ represents the adaptive learning factor of the *r*_th_ scrounger at *t*.

### 3.2. Principle of Extreme Learning Machine

Extreme learning machine (ELM) is based on the feedforward neural network, which is often used to solve the problems of unsupervised learning and supervised learning [50, 51]. The structure of ELM model includes the hidden layer, the output layer, and the input layer. Let *S* be samples, *E* be the input matrix, and *C* be the output matrix. *e* and *c* represent interior parameters of *E* and *C,* respectively. *m* × *S* denotes that *E* is a sample set matrix with *m*-row and *S*-column. *n* × *S* represents that *C* is an *n*-row and *S*-column matrix.

(13) $$\begin{array}{c} E={\left[\begin{array}{cccc} e_{11} & e_{12} & \cdots & e_{1S}\\ e_{21} & e_{22} & \cdots & e_{2S}\\ \cdot & \cdot & \cdots & \cdot \\ e_{m1} & e_{m2} & \cdots & e_{mS} \end{array}\right]}_{m\times S},\\ C={\left[\begin{array}{cccc} c_{11} & c_{12} & \cdots & c_{1S}\\ c_{21} & c_{22} & \cdots & c_{2S}\\ \cdot & \cdot & \cdots & \cdot \\ c_{n1} & c_{n2} & \cdots & c_{nS} \end{array}\right]}_{n\times S},\\ \alpha ={\left[\begin{array}{cccc} \alpha_{11} & \alpha_{12} & \cdots & \alpha_{1m}\\ \alpha_{21} & \alpha_{22} & \cdots & \alpha_{2m}\\ \cdot & \cdot & \cdots & \cdot \\ \alpha_{l1} & \alpha_{l2} & \cdots & \alpha_{lm} \end{array}\right]}_{l\times m}, \end{array}$$

where *α* is the connection weight that connects the input layer and the hidden layer and *l* × *m* indicates that *α* is a matrix with *l* rows and *m* columns.

(14) $$\begin{array}{c} \beta ={\left[\begin{array}{cccc} \beta_{11} & \beta_{12} & \cdots & \beta_{1n}\\ \beta_{21} & \beta_{22} & \cdots & \beta_{2n}\\ \cdot & \cdot & \cdots & \cdot \\ \beta_{l1} & \beta_{l2} & \cdots & \beta_{In} \end{array}\right]}_{l\times n}, \end{array}$$

where *β* is the connection weight that connects the input layer and the hidden layer and *l* × *n* indicates that *β* is a matrix with *l* rows and *n* columns.

*γ*=[*γ*_1_, *γ*_2_,…,*γ*_*l*_]_1×*l*_^*T*^ is the threshold of hidden layer. *α* and *γ* will be optimized by IBSA.

The activation function of the hidden layer is sigmoid function *f*(*x*) as follows:

(15) $$\begin{array}{c} f\left(x\right)=\frac{1}{1+e^{-x}}. \end{array}$$

*C* represents the output of the network as follows:

(16) $$\begin{array}{c} C={\left[c_{1},c_{2},\ldots ,c_{s}\right]}_{n\times S}. \end{array}$$

The element *c*_*j*_ in *C* is expressed as follows:

(17) $$\begin{array}{c} c_{j}={\left[\begin{array}{c} c_{1j}\\ c_{2j}\\ \vdots \\ c_{nj} \end{array}\right]}_{n\times 1}={\left[\begin{array}{c} \sum_{i=1}^{l}\beta_{i1}F\left(\alpha_{i}e_{j}+\gamma_{i}\right)\\ \sum_{i=1}^{l}\beta_{i2}F\left(\alpha_{i}e_{j}+\gamma_{i}\right)\\ \vdots \\ \sum_{i=1}^{l}\beta_{in}F\left(\alpha_{i}e_{j}+\gamma_{i}\right) \end{array}\right]}_{n\times 1}. \end{array}$$

Equation (17) can be simplified as follows:

(18) $$\begin{array}{c} C^{T}=Q\beta . \end{array}$$

In equation (18), *C*^*T*^ is the transposition of *C*, the output of hidden layer is *Q*, and it is expressed as follows:

(19) $$\begin{array}{c} Q={\left[\begin{array}{cccc} F\left(\alpha_{1}\cdot e_{1}+\gamma_{1}\right) & F\left(\alpha_{2}\cdot e_{1}+\gamma_{2}\right) & \cdots & F\left(\alpha_{l}\cdot e_{1}+\gamma_{l}\right)\\ F\left(\alpha_{1}\cdot e_{2}+\gamma_{1}\right) & F\left(\alpha_{2}\cdot e_{2}+\gamma_{2}\right) & \cdots & F\left(\alpha_{l}\cdot e_{2}+\gamma_{l}\right)\\ \cdot & \cdot & \cdots & \cdot \\ F\left(\alpha_{1}\cdot e_{S}+\gamma_{1}\right) & F\left(\alpha_{2}\cdot e_{S}+\gamma_{2}\right) & \cdots & F\left(\alpha_{l}\cdot e_{S}+\gamma_{l}\right) \end{array}\right]}_{S\times l}. \end{array}$$

Huang et al. proposed the following two theorems [50].

**Theorem 1 .**

Given *S* different samples (*i*_*m*_,*c*_*n*_), among them, **e**_*m*_ = [*e*_*i*1_, *e*_*i*2_,…, *e*_*mS*_]^*T*^ ∈ **R**^*m*^, **c**_*i*_ = [*c*_*i*1_,*c*_*i*2_,…*c*_*in*_]^*T*^ ∈ **R**^*n*^. Given an activation function *F*(·): *R* ⟶ **R**, which is infinitely differentiable in any interval. For ELM with *S* hidden layer neurons, if *w*_*i*_ ∈ **R**^*n*^ and *b*_*i*_ ∈ **R** are assigned arbitrarily, the output matrix *Q* of the hidden layer is reversible and ‖*Qβ* − *C*^*T*^‖=0.

From Theorem 1, if the number of neurons in the hidden layer is equal to the number of samples in the training set, with any *w* and *b*, ELM can approach the training sample value with zero error.

**Theorem 2 .**

Given *S* different samples (*i*_*m*_, *c*_*n*_), Among them, **e**_*m*_ = [*e*_*i*1_, *e*_*i*2_,…, *e*_*mS*_]^*T*^ ∈ **R**^*m*^, **c**_*i*_ = [*c*_*i*1_, *c*_*i*2_,…, *c*_*in*_]^*T*^ ∈ **R**^*n*^. Given an activation function *F*(·): *R* ⟶ **R** and any small positive value *ε* > 0. For ELM with *K* (*K* ≤ *S*) hidden layer neurons, if *w*_*i*_ ∈ **R**^*n*^ and *b*_*i*_ ∈ **R** are assigned arbitrarily, then ‖*Qβ* − *C*^*T*^‖ < *ε*.

If the training set number of samples *S* is large, the neurons number in the hidden layer *K* is usually smaller than *S*. It implies that the training error of the ELM can approach any small positive value *ε* > 0.

The *w* and *b* can be randomly selected before training, and they remain unchanged during training. The connection weight *β* between the hidden layer and the input layer can be obtained by solving the least square solution as

(20) $$\begin{array}{c} \underset{\beta }{\min}\left\|Q\beta -C^{T}\right\|. \end{array}$$

From equation (20), $\hat{\beta }=Q^{+}C^{T}$ can be obtained, where *Q*^+^ is the Moore–Penrose generalized inverse of matrix *Q*.

### 3.3. Hybrid Improved Bird Swarm Algorithm with Extreme Learning Machine

During the ELM training process, the number of nodes in the hidden layer of the ELM may increase when the threshold of the hidden layer and the weight of the input layer are not appropriately adjusted. As a result, the prediction stability worsened. Consequently, the improved bird swarm optimization algorithm (IBSA) was involved to obtain optimal solutions for the hidden layer threshold *γ* and input weight *α* in ELM.

**Figure 1:**
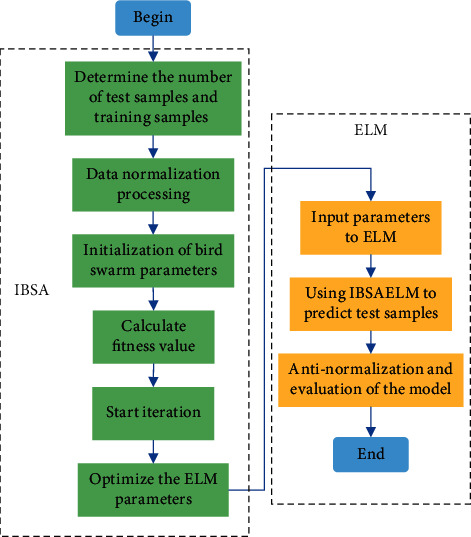
The process of PV power output prediction using the IBSAELM model.

The process of PV power output prediction using IBSAELM model is shown in Figure 1. The specific steps are illustrated as follows: (1) Determining the ratio of training samples and test samples. (2) Normalizing the training samples, i.e., converting the original data to a specific range of values. (3) Initializing the parameters of the BSA, such as population *M*, maximum number of iterations *t*_max_, and migration frequency FQ. (4) Calculating the fitness value and further determining the local optimal individual and the global optimal individual. (5) Starting the iteration training process to optimize the threshold and the weights of the ELM model. At the end of the iteration, the optimal parameters of the ELM model would be obtained. (6) Inputting the optimal parameters to the ELM model. (7) Predicting the PV power using the trained IBSAELM model. (8) Performing out the antinormalization process to restore the normalized value to the original range, and evaluating the prediction effectiveness.

## 4. Performance Results

### 4.1. Effect of Weather Conditions on PV Output Power

**Figure 2:**
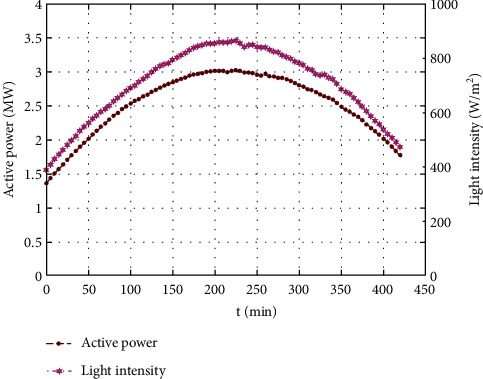
PV output power and light intensity in sunny weather.

**Figure 3:**
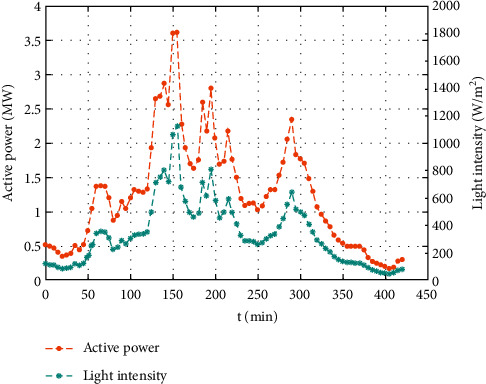
PV output power and light intensity in cloudy weather.

It is well known that depending upon the weather conditions, light intensity is the key factor in PV power output. The relationship between output power and light intensity in sunny weather and cloudy weather is shown in Figures 2 and 3, respectively. The data were collected every 5 min, from 9:00 to 16:00 from the open-access provided by the Desert Knowledge Australia Solar Center. The data for the sunny weather were collected on August 8, 2017, while the data for cloudy weather were collected on October 7, 2017.

As shown in Figures 2 and 3, irrespective of the weather conditions being sunny or cloudy, the PV power output generated is consistent with the light intensity, confirming a positive correlation. Additionally, the light intensity does not vary abruptly from time to time when the weather is sunny. However, the light intensity may fluctuate at any time in cloudy weather. Hence, it is obvious that the condition of the weather has a significant impact on the power output of the PV system.

### 4.2. Convergence Comparison Using Different Models

This section investigates the convergence effect in the IBSA, CSO, BSA, ACO, and PSO algorithms [42, 44, 45]. The basic parameters of IBSA are maximum iterations number *t*_max_=500, the migration frequency of a flock of birds FQ=5, population quantity *M*=30, *e*_1_=1.5, and *e*_2_=1.5. The test function *f*_1_=∑_*i*=1_^*m*^*x*_*i*_^2^ is set in the range of [−100,100] and the optimal value of the function is set as 0. The test function *f*_2_=∑_*i*=1_^*m*^|*x*_*i*_|+∏_*i*=1_^*m*^|*x*_*i*_| is set in the range of [−10,10] and the optimal value of the function is set as 0. The test function *f*_3_=∑_*i*=1_^*m*^(*x*_*i*_^2^ − 10 × cos(2*πx*_*i*_)+10) is set in the range of [−5.12, 5.12] and the optimal value of the function is set as 0. Under the same standard functions as mentioned above, the CSO, PSO, BSA, and IBSA algorithms were tested 15 times with the test dimension = 50. The results are presented in Table 1. Note that the evaluation of all algorithms throughout this study was performed using the MATLAB software, the source code for which can be freely accessed at https://drive.google.com/drive/folders/1h2ZEvBInO7DdAWoaguV96S8KteIfkNw6?usp=sharing.

In function *f*_1_, the IBSA achieved the lowest average convergence value, while the PSO obtained has a value much higher than others. Similarly, in function *f*_2_, the IBSA algorithm exhibited the highest precision, while the PSO obtained relatively worse results than others. In function *f*_3_, both IBSA and BSA algorithms reached a zero-convergence value, i.e., the optimal solution, hence showing better performance than PSO, ACO, and CSO. In conclusion, IBSA exhibited the most favorable convergence effect in functions *f*_1_, *f*_2_, and *f*_3_. In addition, the convergence time of BSA and IBSA was significantly lesser than that of PSO, ACO, and CSO. The convergence time of IBSA was even shorter than that of BSA.

**Figure 4:**
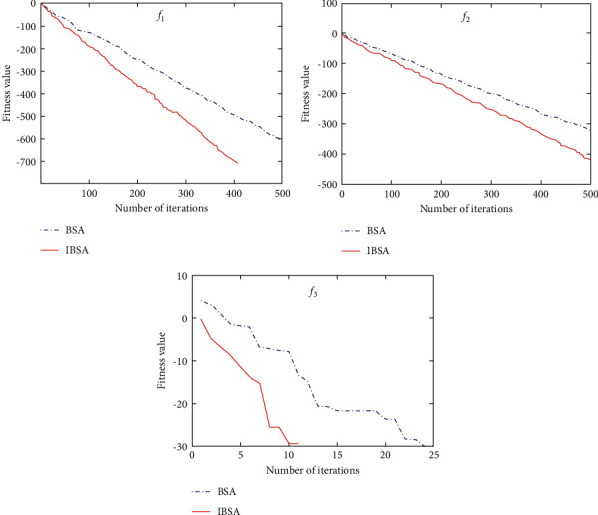
The convergence curve of BSA and IBSA.

The convergence curve of BSA and IBSA using the fitness value is shown in Figure 4; this revealed that the convergence rate of IBSA is faster than that of BSA.

### 4.3. Evaluation of Model Performance

In this study, the sample data for the sunny days were collected from August 8, 2017, to August 12, 2017. The data for the cloudy days were collected from October 6, 2017, to October 10, 2017. The collection process took place every 5 min between 9:00 AM and 6:00 PM every day. Amongst all available data, the data from August 8, 2017, to August 11, 2017, and from October 6, 2017, to October 9, 2017, were chosen to be the training samples. The data from August 12, 2017, to October 10, 2017, were selected as the testing samples. The inputs for the forecast models were relative humidity, temperature, and light intensity, where the output of the forecast model was PV output power. The root mean squared error (RMSE) and the determination coefficient (*R*^2^) were used as evaluation indexes [52]:

(21) $$\begin{array}{c} \text{RMSE}=\sqrt{\frac{1}{m}\sum_{i=1}^{m}{\left(t_{i}^{\wedge }-t_{i}\right)}^{2}},\\ R^{2}=\frac{{\left(m\sum tt^{\wedge }-\sum t\sum t^{\wedge }\right)}^{2}}{\left(m\sum {\left(t^{\wedge }\right)}^{2}-\sum {\left(t^{\wedge }\right)}^{2}\right)\left(m\sum {\left(t\right)}^{2}-\sum {\left(t\right)}^{2}\right)}, \end{array}$$

where *m*, *t*^∧^, and *t* represent the samples number, the predicted value, and the actual value, respectively, and *t*_*i*_^∧^ and *t*_*i*_ indicate the *i*^th^ predicted value and actual value, respectively. The closer the determination coefficient (*R*^2^) is to 1, the better the performance the fitting effect achieves.

**Figure 5:**
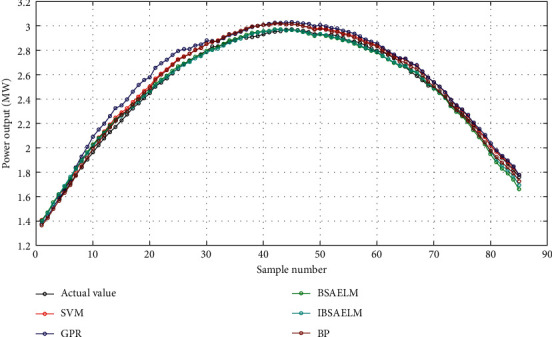
Prediction curves on August 12, 2017, in sunny weather.

**Figure 6:**
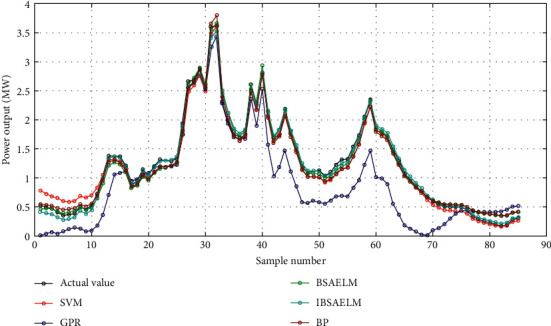
Prediction curves on October 10, 2017, in cloudy weather.

In this study, five models were used to evaluate the effectiveness of PV output power prediction: (1) SVM model. (2) GPR model. (3) BSAELM model. (4) IBSAELM model. (5) BP model. The prediction results from models in sunny and cloudy weather are shown in Figures 5 and 6, respectively.

As shown in Figure 5, the prediction curves of the SVM, BP, GPR, BSAELM, and IBSAELM models are equipped with high fitting precision, due to stable sunlight strength, on sunny days. Subsequently, BSAELM and IBSAELM models exhibited relatively more desirable results.

In Figure 6, in the initial prediction period, the forecast fitting curves of the SVM and GPR models are inferior to others. In the mid-term period, the prediction curve of GPR deviates from the actual output power curve considerably. In the final period, the prediction curves of GPR, BP, and BSAELM models depart from the actual output power curve. On the other hand, the IBSAELM model exhibited a good fitting effect during the whole period.

The absolute error (AE) is defined by Δ*x*=*x* − *x*_*o*_, where *x* is the true value of a quantity, *x*_*o*_ is the measured value, and Δ*x* is the absolute error.

**Figure 7:**
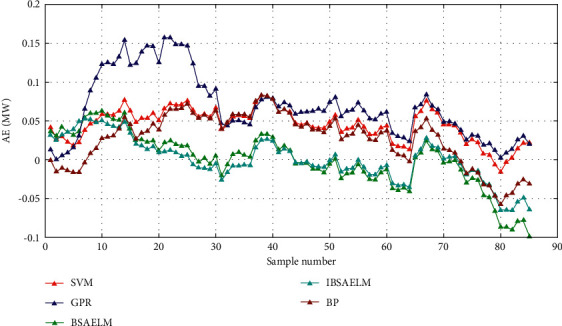
Prediction AE in sunny weather.

The AE using BSAELM, IBSAELM, GPR, BP, and SVM models in sunny weather are shown in Figure 7, where the unit is MW. As can be observed, the output power prediction errors from the five models fell within [−0.1, 0.2]. The highest error beyond 0.15 appeared in the early period while using GPR. However, most forecast errors are located between [−0.1, 0.1]. In the middle period, the prediction errors obtained using BSAELM and IBSAELM were smaller than those from GPR, BP, and SVM, which were located between [−0.05, 0.05]. In the last period, the prediction error from BSAELM reached the largest value, i.e., −0.1. Using IBSAELM resulted in the smallest error value during the whole prediction period and was located between [−0.05, 0.05].

**Figure 8:**
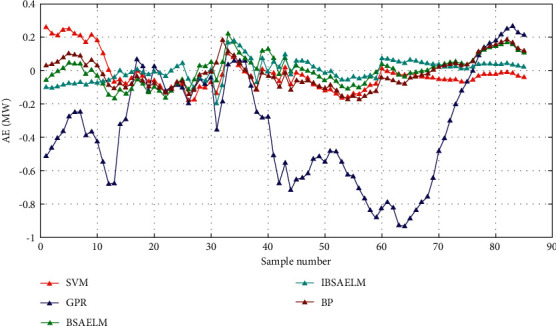
Prediction AE in cloudy weather.

The prediction AE in cloudy weather is shown in Figure 8. In the initial prediction period, the errors from using GPR and SVM models were larger than others. It can be observed that the largest prediction error of the SVM model exceeded 0.2 and that of the GPR model was over 0.6. In the middle prediction period, the errors obtained from the BP, SVM, BSAELM, and IBSAELM were relatively small, remaining between [−0.2, 0.2]. In the last prediction period, the errors using BSAELM and IBSAELM remained relatively small. It was discovered that the error of IBSAELM lied between [−0.2, 0.2], which is the smallest value among the five prediction models, hence proving its suitability to provide showing the most desirable prediction effect.

As a result, it is concluded that the prediction errors in cloudy weather are relatively higher than those in sunny weather. Moreover, the IBSAELM model achieves the lowest prediction error as compared to the other four models.

The evaluation results using RMSE and *R*^2^ are summarized in Table 2.

As presented in Table 2, the prediction result obtained during the sunny days is considerably better than that obtained during cloudy days, for every model using RMSE. For example, the RMSE value of IBSAELM is 2.83 on sunny days and 6.43 on cloudy days. The RMSE values of BP, SVM, GPR, and BSAELM were 4.32, 4.96, 8.03, and 3.64, respectively, on sunny days. However, its respective RMSE values on cloudy days were 9.36, 10.63, 47.20, and 8.58, respectively. Hence, it can be observed that the RMSE of IBSAELM presents the best performance. In contrast, the *R*^2^ of every model reached above 99% on sunny days; however, the *R*^2^ in cloudy weather for each model was relatively lower than that of sunny days. Among all models, only IBSAELM achieved 99% plus on both sunny and cloudy days.

## 5. Conclusions

In this study, the superiority of PV power prediction using the IBSAELM model, under two kinds of weather conditions against four existing models, was verified. The *R*^2^ values obtained from the IBSAELM model remained over 99% and the RMSE values were 2.83 and 6.43 for sunny and cloudy weather, respectively; this indicates that the errors obtained using the IBSAELM model are lower than other models. However, the prediction error obtained in cloudy weather was relatively higher than the error obtained in sunny weather, in all models. The major contributions of this study are concluded as follows:

1. The IBSA algorithm has effectively enhanced the searching ability of the BSA algorithm, by introducing adaptive learning coefficient and factors into the BSA algorithm.
2. The IBSA algorithm efficiently optimizes the ELM model, which improves the prediction ability of the ELM. The IBSAELM model was thus constructed by integrating IBSA and ELM models to accurately predict the PV power.
3. As compared to the SVM, BP, GPR, BSAELM, and IBSAELM models achieve the lowest RMSE value, indicating the best prediction accuracy. Similarly, the *R*^2^ value of IBSAELM in both sunny and cloudy weather reaches over 99%, i.e., higher than that of the other models.

## Figures and Tables

**Table 1:** Convergence value and time of BSA, IBSA, PSO, ACO, and CSO algorithms.

Function	Algorithm	Optimal convergence value	Worst convergence value	Average convergence value	Average time
*f*_1_	BSA	1.06*e* − 265	4.69*e* − 231	1.56*e* − 232	0.062500
	IBSA	8.42*e* − 297	2.11*e* − 271	4.21*e* − 273	0.046875
	PSO	7.94	31.63	17.47	0.167969
	ACO	1.6212*e* − 09	8.6225*e* − 09	3.9054*e* − 09	0.179541
	CSO	4.17*e* − 11	6.34*e* − 05	2.61*e* − 06	0.124063

*f*_2_	BSA	1.93*e* − 133	6.71*e* − 122	3.47*e* − 123	0.093750
	IBSA	1.94*e* − 149	9.81*e* − 133	5.21*e* − 134	0.062500
	PSO	17.30	30.54	23.51	0.145867
	ACO	2.0573*e* − 05	4.1639	0.8952	0.197693
	CSO	7.16*e* − 17	1.57*e* − 11	1.68*e* − 12	0.138750

*f*_3_	BSA	0	0	0	0.125000
	IBSA	0	0	0	0.109375
	PSO	215.56	361.31	283.64	0.160078
	ACO	8.9546	39.7982	18.6389	0.236877
	CSO	6.85*e* − 11	1.36*e* − 2	4.52*e* − 04	0.133437

**Table 2:** Evaluation results of model prediction.

Weather	Model	RMSE	*R*^2^ (%)
Sunny	SVM	4.96	99.81
	GPR	8.03	99.17
	BP	4.32	99.72
	BSAELM	3.64	99.31
	IBSAELM	2.83	99.59

Cloudy	SVM	10.63	98.42
	GPR	47.20	83.48
	BP	9.36	98.78
	BSAELM	8.58	98.83
	IBSAELM	6.43	99.35

## Data Availability

The data that support the findings of this study are openly available from the Desert Knowledge Australia Solar Center at http://dkasolarcentre.com.au/.

## References

[1] Xiong P.-P., Yan W.-J., Wang G.-Z., Pei L.-L. (2019). Grey extended prediction model based on IRLS and its application on smog pollution. *Applied Soft Computing*.

[2] Wang K., Qi X., Liu H. (2019). Photovoltaic power forecasting based LSTM-convolutional Network. *Energy*.

[3] Zhang K., Deletic A., Bach P. M. (2019). Testing of new stormwater pollution build-up algorithms informed by a genetic programming approach. *Journal of Environmental Management*.

[4] Jin X. Y., Li M. Y., Meng F. S. (2019). Comprehensive evaluation of the new energy power generation development at the regional level: an empirical analysis from China. *Energies*.

[5] Geng W., Ming Z., Lilin P., Ximei L., Bo L., Jinhui D. (2016). China’s new energy development: status, constraints and reforms. *Renewable and Sustainable Energy Reviews*.

[6] Gu A., Zhou X. (2020). Emission reduction effects of the green energy investment projects of China in belt and road initiative countries. *Ecosystem Health and Sustainability*.

[7] Shafi A., Sharadga H., Hajimirza S. (2020). Design of optimal power point tracking controller using forecasted photovoltaic power and demand. *IEEE Transactions on Sustainable Energy*.

[8] Zhang T., Lv C., Ma F., Zhao K., Wang H., O’Hare G. M. P. (2020). A photovoltaic power forecasting model based on dendritic neuron networks with the aid of wavelet transform. *Neurocomputing*.

[9] Cheng Z., Liu Q., Zhang W. (2019). Improved probability prediction method research for photovoltaic power output. *Applied Sciences*.

[10] Hu K., Cao S., Wang L., Li W., Lv M. (2018). A new ultra-short-term photovoltaic power prediction model based on ground-based cloud images. *Journal of Cleaner Production*.

[11] Li P., Zhou K., Lu X., Yang S. (2020). A hybrid deep learning model for short-term PV power forecasting. *Applied Energy*.

[12] Jung Y., Jung J., Kim B., Han S. (2020). Long short-term memory recurrent neural network for modeling temporal patterns in long-term power forecasting for solar PV facilities: case study of South Korea. *Journal of Cleaner Production*.

[13] Monfared M., Fazeli M., Lewis R., Searle J. (2019). Fuzzy predictor with additive learning for very short-term PV power generation. *IEEE Access*.

[14] Oprea S.-V., Bâra A. (2020). Ultra-short-term forecasting for photovoltaic power plants and real-time key performance indicators analysis with big data solutions. Two case studies—PV Agigea and PV Giurgiu located in Romania. *Computers in Industry*.

[15] Wang F., Xuan Z., Zhen Z., Li K., Wang T., Shi M. (2020). A day-ahead PV power forecasting method based on LSTM-RNN model and time correlation modification under partial daily pattern prediction framework. *Energy Conversion and Management*.

[16] Han S., Qiao Y.-H., Yan J., Liu Y.-Q., Li L., Wang Z. (2019). Mid-to-long term wind and photovoltaic power generation prediction based on copula function and long short term memory network. *Applied Energy*.

[17] Zhou H., Zhang Y., Yang L., Liu Q., Yan K., Du Y. (2019). Short-term photovoltaic power forecasting based on long short term memory neural network and attention mechanism. *IEEE Access*.

[18] Li L.-L., Wen S.-Y., Tseng M.-L., Chiu A. S. F. (2020). Photovoltaic array prediction on short-term output power method in centralized power generation system. *Annals of Operations Research*.

[19] Li K., Wang F., Mi Z., Fotuhi-Firuzabad M., Duić N., Wang T. (2019). Capacity and output power estimation approach of individual behind-the-meter distributed photovoltaic system for demand response baseline estimation. *Applied Energy*.

[20] Theocharides S., Makrides G., Livera A., Theristis M., Kaimakis P., Georghiou G. E. (2020). Day-ahead photovoltaic power production forecasting methodology based on machine learning and statistical post-processing. *Applied Energy*.

[21] Al-Dahidi S., Ayadi O., Adeeb J., Alrbai M., Qawasmeh B. R. (2018). Extreme learning machines for solar photovoltaic power predictions. *Energies*.

[22] VanDeventer W., Jamei E., Thirunavukkarasu G. S. (2019). Short-term PV power forecasting using hybrid GASVM technique. *Renewable Energy*.

[23] Pazikadin A. R., Rifai D., Ali K., Malik M. Z., Abdalla A. N., Faraj M. A. (2020). Solar irradiance measurement instrumentation and power solar generation forecasting based on artificial neural networks (ANN): a review of five years research trend. *The Science of the Total Environment*.

[24] Wang H., Liu Y., Zhou B. (2020). Taxonomy research of artificial intelligence for deterministic solar power forecasting. *Energy Conversion and Management*.

[25] Lee J., Zhang P., Gan L. K. (2018). Optimal operation of an energy management system using model predictive control and Gaussian process time-series modeling. *IEEE Journal of Emerging and Selected Topics in Power Electronics*.

[26] Sorkun M. C., Durmaz Incel Ö., Paoli C. (2020). Time series forecasting on multivariate solar radiation data using deep learning (LSTM). *Turkish Journal of Electrical Engineering and Computer Sciences*.

[27] Gao R. B., Duru O. (2020). Parsimonious fuzzy time series modelling. *Expert Systems with Applications*.

[28] Sharadga H., Hajimirza S., Balog R. S. (2020). Time series forecasting of solar power generation for large-scale photovoltaic plants. *Renewable Energy*.

[29] Dawan P., Sriprapha K., Kittisontirak S. (2020). Comparison of power output forecasting on the photovoltaic system using adaptive neuro-fuzzy inference systems and particle swarm optimization-artificial neural network model. *Energies*.

[30] Deo R. C., Şahin M. (2017). Forecasting long-term global solar radiation with an ANN algorithm coupled with satellite-derived (MODIS) land surface temperature (LST) for regional locations in Queensland. *Renewable and Sustainable Energy Reviews*.

[31] Nespoli A., Ogliari E., Leva S. (2019). Day-ahead photovoltaic forecasting: a comparison of the most effective techniques. *Energies*.

[32] Rocha A. S. F., Guerra F. K. d. O. M. V., Vale M. R. B. G. (2020). Forecasting the performance of a photovoltaic solar system installed in other locations using artificial neural networks. *Electric Power Components and Systems*.

[33] Khan I., Zhu H., Yao J., Khan D., Iqbal T. (2017). Hybrid power forecasting model for photovoltaic plants based on neural network with air quality index. *International Journal of Photoenergy*.

[34] Sheng H., Xiao J., Cheng Y., Ni Q., Wang S. (2018). Short-term solar power forecasting based on weighted Gaussian process regression. *IEEE Transactions on Industrial Electronics*.

[35] Bae K. Y., Jang H. S., Sung D. K. (2016). Hourly solar irradiance prediction based on support vector machine and its error analysis. *IEEE Transactions on Power Systems*.

[36] Liu M. S., Cao Z. M., Zhang J., Wang L., Huang C., Luo X. (2020). Short-term wind speed forecasting based on the jaya-SVM model. *International Journal of Electrical Power & Energy Systems*.

[37] Wang F., Zhen Z., Wang B., Mi Z. (2018). Comparative study on KNN and SVM based weather classification models for day ahead short term solar PV power forecasting. *Applied Sciences*.

[38] Aprillia H., Yang H. T., Huang C. M. (2020). Short-term photovoltaic power forecasting using a convolutional neural network-salp swarm algorithm. *Energies*.

[39] Yang S. X., Zhu X. G., Peng S. J. (2020). Prospect prediction of terminal clean power consumption in China via LSSVM algorithm based on improved evolutionary game theory. *Energies*.

[40] Hossain M., Mekhilef S., Danesh M., Olatomiwa L., Shamshirband S. (2017). Application of extreme learning machine for short term output power forecasting of three grid-connected PV systems. *Journal of Cleaner Production*.

[41] Zhou Y., Zhou N. R., Gong L. H., Jiang M. L. (2020). Prediction of photovoltaic power output based on similar day analysis, genetic algorithm and extreme learning machine. *Energy*.

[42] Liu Z.-F., Luo S.-F., Tseng M.-L. (2021). Short-term photovoltaic power prediction on modal reconstruction: a novel hybrid model approach. *Sustainable Energy Technologies and Assessments*.

[43] Luo X., Sun J., Wang L. (2018). Short-term wind speed forecasting via stacked extreme learning machine with generalized correntropy. *IEEE Transactions on Industrial Informatics*.

[44] Li L. L., Liu Z. F., Tseng M. L., Zheng S. J., Lim M. K. (2021). Improved tunicate swarm algorithm: solving the dynamic economic emission dispatch problems. *Applied Soft Computing*.

[45] Li L. L., Zhao X., Tseng M. L., Tan R. R. (2020). Short-term wind power forecasting based on support vector machine with improved dragonfly algorithm. *Journal of Cleaner Production*.

[46] Meng X.-B., Gao X. Z., Lu L., Liu Y., Zhang H. (2015). A new bio-inspired optimisation algorithm: bird swarm algorithm. *Journal of Experimental & Theoretical Artificial Intelligence*.

[47] Alhassan A. M., Wan Zainon W. M. N. (2020). Taylor bird swarm algorithm based on deep belief network for heart disease diagnosis. *Applied Sciences*.

[48] Huang C., Sheng X. (2020). Data-driven model identification of boiler-turbine coupled process in 1000 MW ultra-supercritical unit by improved bird swarm algorithm. *Energy*.

[49] Li L. L., Liu Y. W., Tseng M. L., Lin G. Q., Ali M. H. (2020). Reducing environmental pollution and fuel consumption using optimization algorithm to develop combined cooling heating and power system operation strategies. *Journal of Cleaner Production*.

[50] Huang G.-B., Zhu Q.-Y., Siew C.-K. (2006). Extreme learning machine: theory and applications. *Neurocomputing*.

[51] Wu X., Lai C. S., Bai C., Lai L. L., Zhang Q., Liu B. (2020). Optimal kernel ELM and variational mode decomposition for probabilistic PV power prediction. *Energies*.

[52] Huang C.-J., Kuo P.-H. (2019). Multiple-input deep convolutional neural network model for short-term photovoltaic power forecasting. *IEEE Access*.

